# Association between metabolic parameters and glomerular hyperfiltration in a representative Korean population without chronic kidney disease

**DOI:** 10.1371/journal.pone.0207843

**Published:** 2018-12-06

**Authors:** Sangmo Hong, Yun Mi Choi, Sung-Hee Ihm, Dooman Kim, Moon-Gi Choi, Jae Myung Yu, Eun-Gyoung Hong

**Affiliations:** 1 Division of Endocrinology and Metabolism, Department of Internal Medicine, Hallym University Dongtan Sacred Heart Hospital, Hwaseong, Korea; 2 Division of Endocrinology and Metabolism, Hallym University Sacred Heart Hospital, Hallym University College of Medicine, Gyeonggi-do, Republic of Korea; 3 Department of Endocrinology and Metabolism, Division of Internal Medicine, Kangdong Sacred Heart Hospital, Hallym University College of Medicine, Seoul, Korea; 4 Division of Endocrinology and Metabolism, Department of Internal Medicine, College of Medicine, Hallym University, Chuncheon, Republic of Korea; 5 Division of Endocrinology Metabolism and Geriatric Medicine, Department of Internal Medicine, Hallym University Kangnam Sacred Heart Hospital, Hallym University College of Medicine, Seoul, Korea; University of Colorado Denver School of Medicine, UNITED STATES

## Abstract

**Aims:**

To investigate associations of glomerular hyperfiltration with other metabolic factors in a nationally representative dataset.

**Methods:**

We analyzed cross-sectional data from 15,918 subjects with estimated glomerular filtration rate (eGFR) >60 ml/min/1.73 m^2^ and urine albumin creation ratio (ACR) <30 mg/g, who participated in the 5th and 6th Korea National Health and Nutrition Examination Surveys. Hyperfiltration was defined as eGFR (CKD-EPI equation) exceeding the age- and sex-specific 95th percentile for healthy control subjects.

**Results:**

Prevalence of hyperfiltration was 5.2% and that among normal, prediabetic, and diabetic subjects was 4.9%, 5.6%, and 7.3%, respectively, after adjusting for age, sex, and body weight (p for trend = 0.008). In a multiple logistic regression analysis, hyperfiltration was associated with a body mass index ≥30 kg/m^2^ [odds ratio (OR) = 3.461, p<0.001], waist circumference 85 cm (men) or 80 cm (women) (OR = 1.425, p = 0.015), systolic blood pressure 120–129 mmHg (OR = 1.644, p = 0.022), fasting plasma glucose 140 mg/dL (OR = 1.695, p = 0.033) and t serum triglyceride level 500 mg/dL (OR = 2.988, p = 0.001), and was independently associated with the ACR (B = 0.053, p<0.001).

**Conclusions:**

In a general Korean population, both hyperfiltration and ACR were associated with similar metabolic parameters, and hyperfiltration correlated independently with a high ACR. Longitudinal studies are needed to further explore risks of hyperfiltration and microalbuminuria.

## Introduction

Glomerular hyperfiltration (GH) has been defined either as an abnormally high whole-kidney glomerular filtration rate (GFR), increased filtration fraction, or increased filtration per nephron[1]. GFR is regulated by renal blood flow, the hydraulic pressure gradient across the kidney membrane and the ultrafiltration coefficient. Previous studies showed that GH is characterized by increasing GFR with increasing generation of nitric oxide, renal kallikrein and other vasoactive kinins[2]. Although GH may occur in otherwise healthy people in response to high protein loading [3] and pregnancy or in patients with various diseases (e.g., obesity, polycystic kidney disease, and sickle cell anemia), it is a frequent diabetic complication that affects around 70% and 50% of patients with type 1 and 2 diabetes, respectively, at diagnosis or during the first few years of the disease [1, 4]. Experimental diabetes models suggested that GH is mediated by increased proximal tubular reabsorption of glucose and sodium, which causes vasodilation secondary to suppressed tubuloglomerular feedback[5]. GH is considered an important factor in the initiation of glomerular damage, especially in diabetic patients [6, 7] and is believed to be a sign of intraglomerular hypertension leading to albuminuria[8].

At present, GH is defined as an abnormal absolute increase in the GFR [9]. Although a consensus has not yet been reached, conventionally, GH has been defined as a GFR of >2 standard deviations above the mean GFR of healthy individuals [1] or above the age- and sex-specific 95th percentile corresponding to the subject [10]. A recent systemic review to clear the definition of GH with 405 studies reported that the thresholds for GH had ranged from 90.7 to 175 ml/min/1.73 m^2^ (median, 135 ml/min per 1.73 m^2^) [11]; of these studies, 30% did not justify the selected threshold value and most remaining studies did not report an appropriate age- and sex-adjusted control group [11]. Furthermore, although the development of GH can precede a diagnosis of diabetes, most previous studies included only patients with already diagnosed diabetes or obesity.

The above-stated inconsistencies among studies, use of limited populations, and lack of agreement regarding an appropriate definition have led to many remaining uncertainties regarding GH. Therefore, additional population-based studies are needed to define the prevalence and characteristics of GH. In this study, we evaluated the potential relationships of GH with various metabolic parameters in a general population without chronic kidney disease, using a dataset from the Korea National Health and Nutrition Examination Survey (KNHANES), which is nationally represents the Korean population.

## Methods

### Participants

The Korea National Health and Nutrition Examination Survey (KNHANES), a nationwide cross-sectional survey representative of the non-institutionalized civilian population in Korea, is conducted periodically by the Korea Centers for Disease Control and Prevention (KCDC). The KNHANES was initiated in 1998 to provide comprehensive information about the health status, health behavior, and nutritional status of the Korean population [12]. To ensure an unbiased national estimate, a sample weight designated by the KCDC to account for the complex survey design, survey non-response, and post-stratification was assigned to the participating individuals [12]. All participants in the KNHANES signed an informed consent form, and the survey was approved by the institutional review board of the KCDC (IRB No. 2011-02CON-06-C). Accordingly, the current study is exempt from the requirement for informed consent because the design involves a secondary analysis of a public dataset where individuals are not identifiable.

For the present study, we acquired data from 32,144 subjects who participated in the 5th (V-2,3, 2011–20012) and 6th (VI-1,2, 2013–2014) KNHANES. From this population, we excluded 6898 subjects younger than 18 years, 7,278 subjects with incomplete data, 67 subjects who were pregnant, 463 subjects without 8-hour fasting data, 712 subjects with an estimated glomerular filtration rate (eGFR) <60 ml/min/1.73 m^2^, 138 subjects with albuminuria [urine albumin creatinine ratio (ACR) ≥30 mg/g], and 670 subjects with a history of cancer. Finally, our analysis included data from 15,918 subjects. Subjects with a previous confirmed diagnosis of diabetes, those taking insulin or oral hypoglycemic agents, or fasting plasma glucose (FPG) ≥126 mg/dL and/or glycated hemoglobin (HbA1ᴄ) ≥6.5% were classified as having diabetes (n = 1553, 9.8%). Prediabetes was classified as FPG between 100 and 125 mg/dL and/or HbA1c between 5.7% and 6.4% (n = 3385, 21.3%). The rest were classified as normal glucose tolerance (n = 10980, 68.9%).

### Measures

Blood samples were collected after an 8-hour fasting period and immediately processed, refrigerated, and transported in cold storage to a central laboratory (NeoDin Medical Institute, Seoul, South Korea) for analysis within 24 hours. The glucose level was measured using the hexokinase method. Serum total cholesterol and triglyceride levels were measured using an enzymatic method. High and low-density lipoprotein cholesterol levels were measured using a homogeneous enzymatic colorimetric method. The above-listed biochemical markers were analyzed using a Hitachi Automatic Analyzer 7600 (Hitachi High-Technologies Co., Tokyo, Japan). HbA1c was measured via high-performance liquid chromatography on an HLC-723G7 device (Tosoh Corporation, Tokyo, Japan). Nutrient intakes, including total calorie, carbohydrate, fat, protein, and sodium intakes, were assessed by a trained dietitian through face-to-face interviews in the participants' homes and by 24-hour dietary recall questionnaires. The results were calculated using the Food Composition Table developed by the National Rural Resources Development Institute (7th revision) [12]. Alcohol consumption was defined as consumption of alcoholic beverages ≥2 times per month within the previous year.

### Estimated glomerular filtration rate (eGFR) and hyperfiltration definition

Serum creatinine concentration was measured using the Jaffe method, rate-blanked, and compensated using a Hitachi Automatic Analyzer 7600. The estimated GFR (eGFR) was calculated using the CKD-EPI equation as follows [13]: for women, GFR = 144 × (Serum creatinine/0.7)^-0.329^ × (0.993)^Age^ with a serum creatinine level ≤0.7 mg/dL and 144 × (Serum creatinine/0.7)^-1.209^ × (0.993)^Age^ with a level >0.7 mg/dL; for men, GFR = 141 × (Serum creatinine/0.9)^-0.411^ × (0.993)^Age^ with a serum creatinine level ≤0.9 mg/dL and 141 × (Serum creatinine/0.9)^-1.209^ × (0.993)^Age^ with a level >0.9 mg/dL. GH was defined as an eGFR exceeding the age- and sex-specific 95th percentile for healthy subjects (n = 5,907), which were defined by excluding subjects with prediabetes (n = 3,385), diabetes (n = 1,553), and prehypertension or hypertension (n = 5073) from the analysis set (n = 15,918). Then we divided these healthy subjects into 10-year age groups and obtained the age group and sex specific eGFR cut-off (above the 95^th^ percentile) for GH (S1 and S2 Tables)[14].

### Statistical analysis

Data are presented as means ± standard deviations (SDs) for continuous variables and as percentages for categorical variables. An unpaired t-test, analysis of variance (ANOVA) or χ2 test was used to compare groups. Odds ratios (ORs) and 95% confidence intervals (CIs) for GH were estimated using a logistic regression analysis adjusted for age and sex (adjusted ORs) and for age, sex, and body weight, body mass index, waist circumference, alcohol drink, smoking, systolic blood pressure, fasting plasma glucose, serum triglyceride, energy intake, and antihypertensive and lipid lowering medication. ORs and 95% CIs for ACR were estimated using a logistic regression analysis adjusted for body weight, body mass index, waist circumference, alcohol drink, smoking, hyperfiltration, systolic blood pressure, fasting plasma glucose, serum triglyceride, energy intake and antihypertensive and lipid lowering medication. All statistical analyses were performed using SPSS version 18.0 for Windows (SPSS Inc., Chicago, IL, USA). P values of <0.05 were considered to indicate statistical significance.

## Results

The 15,918 subjects (men = 6701 and women = 9217) included in this study were divided by the presence or absence of GH. The age-, sex-, and body weight-adjusted characteristics of subjects with or without GH are presented in Table 1. Notably, subjects with GH had a significantly higher BMI (p<0.001), waist circumference (p<0.001), systolic blood pressure (p = 0.001), HbA1c (p = 0.006), FPG(p<0.001), serum triglyceride level (p<0.001), current smoker rate (p<0.001), alcohol consumption (p = 0.018), and antihypertensive and lipid lowering medication use(both p<0.001) compared to those without GH. Although our population was limited to subjects with an ACR <30 mg/g, the ACR value was significantly higher among subjects with GH (p<0.001).ORs of GH increased with higher BMI (p<0.001), waist circumference (p = 0.006), systolic blood pressure (p = 0.066), glucose tolerance (p = 0.024) and t serum triglyceride (p = 0.009) in multiple logistic regression analyses (Table 2). Current smoking (p = 0.007) and alcohol drinking (p = 0.046) were also associated with higher ORs of GH [ORs = 1.393 (95% CI: 1.095–1.773) and ORs = 1.225 (95% CI: 1.004–1.495), respectively] (Table 2).

**Table 1:** Demographic and clinical characteristics of subjects according to hyperfiltration status after adjusting for age, sex, and body weight.

Parameter	Hyperfiltration (-)	Hyperfiltration (+)	
N (%)	15109 (94.9)	809(5.1)	
Age, years	50.08±16.07	46.67±16.43	<0.001
Male gender, (%	42.1	41.8	0.854
BMI, kg/m^2^	23.69±0.01	24.03±0.05	<0.001
Waist circumference, cm	80.78±0.04	82.26±0.16	<0.001
Current smoking, %	18.7	23.8	<0.001
Alcohol consumption, %	48.2	52.8	0.018
SBP, mmHg	118.2±0.1	119.9±0.5	0.001
DBP, mmHg	75.4±0.1	75.1±0.3	0.463
Antihypertensive medication, %	18.9	13.8	<0.001
HbA1c, %	5.772±0.006	5.843±0.025	0.006
Fasting plasma glucose, mg/dL	97.97±0.16	100.49±0.69	<0.001
Diabetes mellitus, %	9.7	10.5	0.401
Diabetes duration, years	8.30±7.52	7.19±7.26	0.240
Antidiabetic medication, %	6.17	7.05	0.314
Insulin therapy, %	0.51	0.49	0.952
Total cholesterol, mg/dL	189.6±0.3	188.5±1.2	0.391
Triglyceride, mg/dL	129.9±0.8	146.8±3.4	<0.001
HDL-cholesterol, mg/dL	52.72±0.10	53.17±0.41	0.291
Lipid lowering medication, %	7.3	3.3	<0.001
Serum creatinine, mg/dL	0.824±0.001	0.622±0.003	<0.001
eGFR, ml/min/1.73 m^2^	94.49±0.08	110.47±0.35	<0.001
Urine creatinine, g/dL	1.543±0.062	1.278±0.267	<0.001
Urine albumin, mg/dL	0.136±0.003	0.163±0.012	0.036
ACR, mg/g	0.951±0.018	1.399±0.079	<0.001
Energy intake, kcal	2019±7	2094±29	0.010
Water intake, g	1064±5	1089±23	0.293
Protein intake, g	71.67±0.32	73.43±1.38	0.215
Fat intake, g	42.18±0.26	42.81±1.12	0.580
Carbohydrate intake, g	319.5±1.0	326.3±4.5	0.215
Sodium intake, mg	4487±25	4579±110	0.421

BMI, body mass index; SBP, systolic blood pressure; DBP, diastolic blood pressure; HbA1c, glycated hemoglobin; HDL, high-density lipoprotein; eGFR, estimated glomerular filtration rate; ACR, albumin creation ratio

**Table 2:** Results of multiple logistic regression analysis with hyperfiltration as the dependent variable adjusted with clinical and metabolic parameters*.

Independent variables	Odd ratio	95% CI	*p*-value
Body mass index, kg/m^2^			0.001
<23	Ref.		
23–24.99	1.073	0.802–1.435	
25–26.99	1.195	0.792–1.804	
27–29.99	1.621	0.976–2.692	
≥30	3.467	1.870–6.428	
Waist circumference, cm			0.006
Men <85, Women <80	Ref.		
Men ≥85, Women ≥80	1.547	1.134–2.110	
Current smoking, %			0.007
No	Ref.		
Yes	1.393	1.095–1.773	
Alcohol consumption, %			0.046
No	Ref.		
Yes	1.225	1.004–1.495	
Systolic blood pressure, mmHg			0.066
<100	1.339	0.948–1.890	
100–109	Ref.		
110–119	1.259	0.931–1.703	
120–129	1.617	1.162–2.249	
≥130	1.321	0.943–1.849	
Status of glucose tolerance			0.024
Normal glucose tolerance	Ref.		
Prediabetes	0.970	0.754–1.249	
Diabetes	1.519	1.094–2.108	
Serum triglyceride, mg/dL			0.009
Normal (<150)	Ref.		
Borderline (150–199)	0.870	0.645–1.174	
High (200–499)	0.949	0.703–1.282	
Very high (≥500)	2.524	1.422–4.479	

CI, confidence interval; Ref., reference

* age, sex, body weight, body mass index, waist circumference, alcohol drink, smoking, systolic blood pressure, fasting plasma glucose, serum triglyceride, energy intake and antihypertensive and lipid lowering medication.

### Hyperfiltration and glucose tolerance

**Fig 1:**
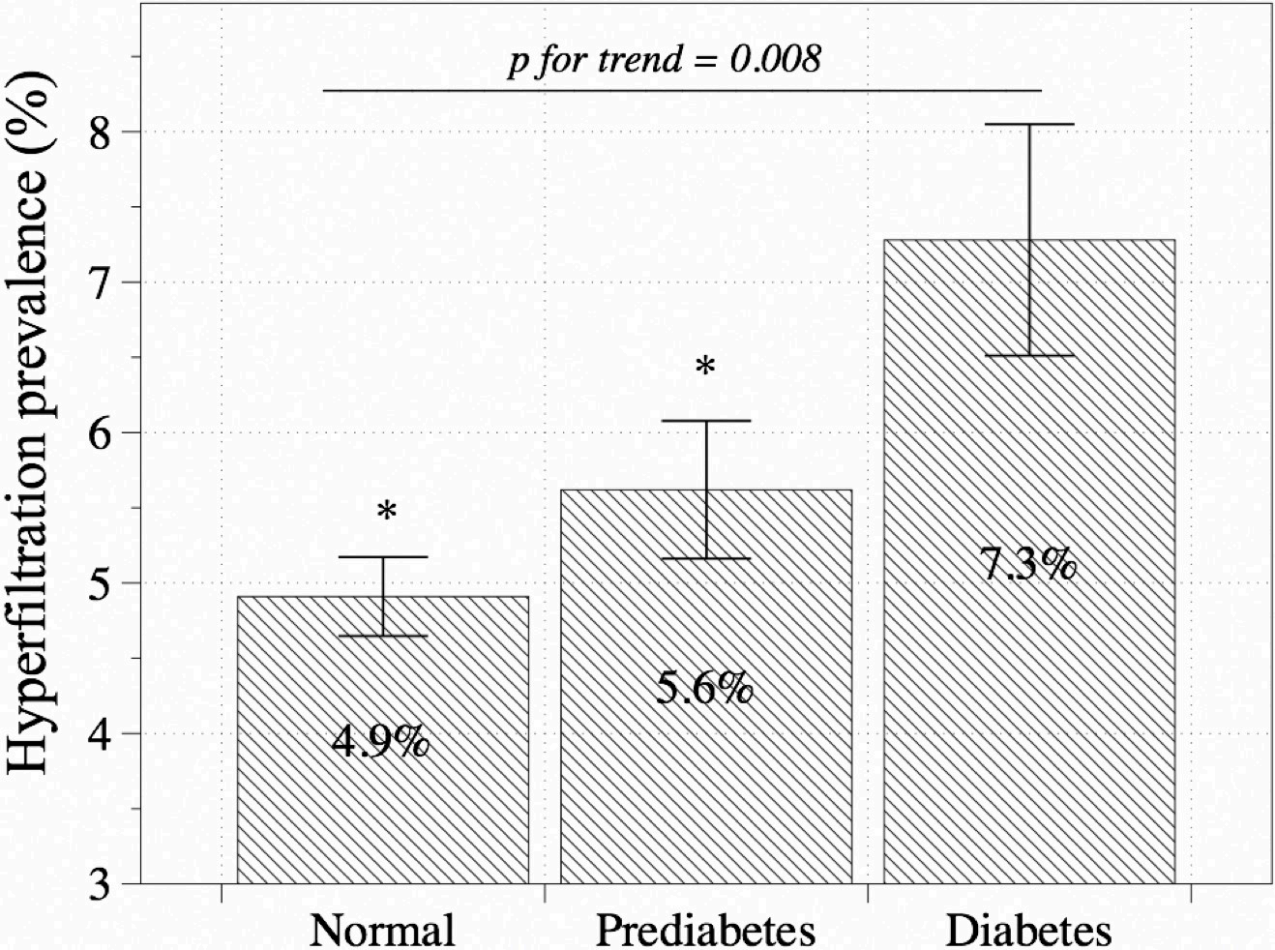
Prevalence of hyperfiltration assessed by status of glucose tolerance. This sample was restricted to subjects older than 18 years who were not pregnant and had no apparent chronic kidney disease. The error bars represent the 95% confidence intervals. * P<0.05 vs. Diabetes group (Bonferroni method).

After adjusting for age, sex and body weight, the prevalence of GH was 4.9% among normal glucose tolerance, 5.6% among prediabetic, and 7.3% among diabetic subjects (*p* for trend = 0.008, Fig 1). In the logistic regression model analyses after adjusting for age, sex, body weight, obesity, current smoking, alcohol consumption, systolic blood pressure, serum triglyceride levels, energy intake and antihypertensive and lipid lowering medication, diabetes had 1.519 (95% CI: 1.094–2.108, p = 0.013) times increased ORs for GH than normal glucose tolerance (Table 2). Besides, the ORs of GH increased as the FPG level increased (S3 Table). The ORs for GH among subjects with FPG ≥126 mg/dL was 1.474 times higher (95% CI: 1.006–2.162, p = 0.047) relative to those with FPG ≤99 mg/dL, and the ORs for GH among subjects with FPG ≥140 mg/dL was 1.980 times higher (95% CI: 1.304–3.006, p = 0.001) relative to those with FPG ≤99 mg/dL, after adjusting for age, sex, body weight, BMI, waist circumference, current smoking, alcohol consumption, systolic blood pressure, serum triglyceride level, energy intake and antihypertensive and lipid lowering medication.

### Hyperfiltration and blood pressure

In our study, subjects with a systolic blood pressure of 100–109 mmHg exhibited the lowest ORs of GH, whereas both below or above this range was associated with a higher ORs of GH (Table 3). Subjects with prehypertension (systolic blood pressure of 120–129 mmHg) had the highest ORs for GH (OR = 1.617, 95% CI: 1.162–2.249). Although subjects with a systolic blood pressure of ≥130 mmHg had a slightly lower ORs of GH when compared to those with prehypertension (OR = 1.321, 95% CI: 0.943–1.849), they presented with significantly higher ACR than those with a systolic blood pressure 120–129 mmHg (mean: 0.668±0.029 vs. 1.430±0.069 mg/g, *p*<0.001).

**Table 3:** Results of clinical and metabolic parameters* adjusted multiple logistic regression analysis with urine albumin creatinine ratio as the dependent variable.

Independent variables	Standardized Coefficients	*p*-value
Hyperfiltration	0.029	<0.001
SBP (mmHg)	0.155	<0.001
Hypertension medication	0.080	<0.001
Fasting plasma glucose (mg/dL)	0.059	<0.001
HbA1c (%)	0.081	<0.001
Triglyceride (mg/dL)	0.052	<0.001

SBP, systolic blood pressure; HbA1c, glycated hemoglobin

* age, sex, body weight, body mass index, waist circumference, alcohol drink, smoking, systolic blood pressure, fasting plasma glucose, serum triglyceride, energy intake and antihypertensive and lipid lowering medication.

### Hyperfiltration and serum triglyceride level

We next stratified the subjects based on the ATP III Classification of serum Triglycerides (Normal: <150, Borderline: 150–199, high: 200–499, Very high: ≥500 mg/dL). Subjects in the very high class had a 2.524-fold increased ORs of GH relative to the normal classes (95% CI: 1.422–4.479 vs. normal class; p<0.001) even after adjusting for age, sex, body weight, BMI, waist circumference, current smoking, alcohol consumption, systolic blood pressure, FPG, energy intake and antihypertensive and lipid lowering medication. A serum triglyceride level ≥400 mg was found to significantly increase the ORs of GH (OR: 1.690, 95% CI: 1.006–2.837, p = 0.047), and an increasing serum triglyceride level was generally found to associate with an increased ORs of GH (S3 Table).

### Interaction between metabolic factors associated with hyperfiltration

**Fig 2:**
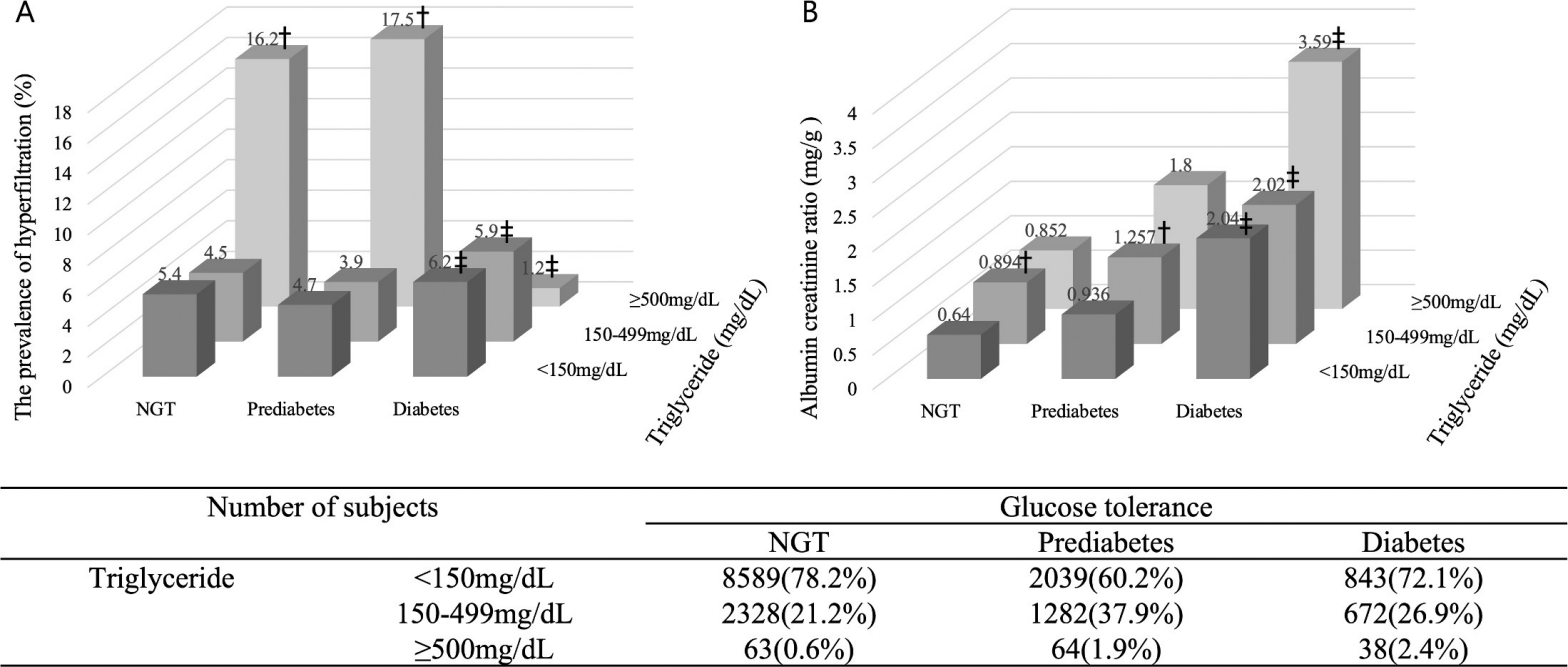
Prevalence of hyperfiltration by serum triglyceride level and diabetes status after adjusting for clinical and metabolic parameters*. NGT, normal glucose tolerance. * age, sex, body weight, body mass index, waist circumference, systolic blood pressure, and energy intake. † P<0.05 vs. a serum triglyceride level <150 mg/dL (Bonferroni method). ‡ P<0.05 vs. Normal glucose tolerance (Bonferroni method).

Fig 2A depicts the prevalence of GH according to serum triglyceride level and diabetes status. Compared to a serum triglyceride level <150 mg/dL, a serum triglyceride level of ≥500 mg/dL was associated with an increased ORs of GH among subjects with normal glucose tolerance and prediabetes (all p< 0.01). Among subjects with a serum triglyceride level below 500 mg/dL, the prevalence of GH increased among subjects with diabetes (5.9% vs. 4.5%, p = 0.030 for those with serum triglycerides 150–499 mg/dL and 6.2% vs. 5.4%, p = 0.013 among those with serum triglycerides <150 mg/dL) than among subjects with normal glucose tolerance. Interestingly, a low prevalence of GH was observed among subjects with diabetes and serum triglyceride level ≥500 mg/dL than subjects with normal glucose tolerance or prediabetes with serum triglyceride level ≥500 mg/dL (p = 0.013); however, these subjects had the highest ACR level (Fig 2). Table 3 presents the results of a multiple regression analysis with ACR as a dependent variable. Hyperfiltration was found to independently associate with ACR (standardized beta coefficients; B = 0.029, p<0.001).

## Discussion

Despite increasing awareness of GH as a precursor to chronic kidney disease, previous studies have only evaluated this factor within specific subpopulations. To address the lack of GH data from a general population, we evaluated the association between GH and other risk factors in an age- and sex-specific analysis of a national representative population dataset (KNHANES). In this population-based study, we found that GH is associated with obesity, hyperglycemia, hypertension, hypertriglyceridemia, alcohol consumption and smoking all of which are also known to associate with diabetes and chronic kidney disease. Interestingly, we found that a serum triglyceride level of ≥500 mg/dL independently increased the ORs of GH by 2.5-fold, compared to a serum triglyceride level <150 mg/dL. Paradoxically, subjects with diabetes and serum triglyceride ≥500 mg/dL had a low prevalence of GH but the highest mean ACR level. Furthermore, GH was independently associated with the ACR level.

Chronic kidney disease (CKD) has become a serious public health issue. The best way to reduce the burden of CKD might be early diagnosis and intervention. Although GH is not established to be a precursor of CKD, it is believed to play an important role in the initiation of glomerular damage. Diabetes and hypertension are well known risks factors for CKD and excessive alcohol consumption, smoking, the use of analgesic medications, and obesity also constitute risks for CKD[15]. Our study showed that the risk factors for GH (obesity, current smoking, alcohol consumption, diabetes rand elevated systolic blood pressure and serum triglyceride) were similar the risk factors of CKD. Although our study was cross-sectional analysis, our finding supports the hypothesis that GH might be a precursor of CKD.

Previously, Calamari et al. proposed GH to be a precursor of the diabetic nephropathy in type 1 diabetes, in which GH is often found to precede changes in albuminuria by several years [16]. However, a somewhat different course has been observed in patients with type 2 diabetes. A well-conducted study of 194 Pima Indians with different stages of glucose tolerance found that the GFR was already elevated at the onset of diabetes; additionally, patients with impaired glucose tolerance had a higher GFR relative to normal controls. These results suggest that hyperfiltration may even precede the onset of diabetes [4], and further suggest that factors other than hyperglycemia may contribute to hyperfiltration. We also observed an increased prevalence of hyperfiltration according to glucose tolerance (Fig 1, p = 0.008), as well as a significant ORs of diabetes (relative to normal glucose tolerance) with GH after adjusting for compounding factors. An FPG higher than 126 mg/dL, which is the accepted cut-off for the diagnosis for diabetes, was lowest significant cut-off for the GH and higher FPG associated with the higher risk for the GH. And these can explain the association between GH and type 2 diabetes, and the role of hyperglycemia in GH.

On the other hands, the association between hypertension and GH was not as well-known as diabetes. Some studies have shown associations among hypertension, GH, and chronic kidney disease. In an animal model of salt-dependent hypertension, GH developed concurrently and was associated with a more rapid GFR decline and glomerular sclerosis [17]. In a previous study of human patients with stage 1 hypertension and without diabetes, those with GH had a 4-fold higher ORs (95% CI: 2.1–7.4, p = 0.001) of developing microalbuminuria during a mean follow-up of 7.8 years [18]. Another study of hypertensive patients demonstrated the early development of GH during sympathetic nervous system activation, which is mediated by postglomerular vasoconstriction [19]. Our study observed the lowest ORs of GH among patients with a systolic blood pressure of 100–109 mmHg, whereas the highest ORs were observed among those with a systolic blood pressure of 120–129 mmHg. The group with the lowest ORs group in our study had systolic blood pressure measurements <120 mmHg, the accepted cut-off for normal systolic blood pressure, whereas the highest ORs group had measurements near this cut-off. This finding suggests that patients with prehypertension should be monitored for GH, although a prospective study is needed to confirm our results.

Few data are available to support or contradict the association between GH and hypertriglyceridemia. A recent study of adolescents aged 12–17 years who participated in the National Health and Nutrition Examination Survey, a nationally representative sample of the US population, found that hypertriglyceridemia (≥110 mg/dL) was associated with a 1.58-fold increased ORs (95% CI: 1.11–2.23) of GH [20]. Still, although some studies have demonstrated an association between hypertriglyceridemia and CKD, a causal relationship has not been defined. In a study of nondiabetic patients with CKD, Samuelsson *et al*. reported an association of triglyceride-rich apo B-containing lipoproteins with the rate of chronic kidney disease progression [21]. In another study, patients with low HDL cholesterol levels and hypertriglyceridemia at baseline had a higher ORs of CKD [22]. Studies involving the FinneDiane (Finnish Diabetic Nephropathy) study had reported that higher serum triglyceride were associated with progressive albuminuria, whereas total cholesterol was associated with progression to ESRD in an adjusted Cox regression model that included HbA1c level, sex, smoking, BP, BMI, duration of diabetes, and eGFR[23]. As noted above, in our study, hypertriglyceridemia was associated with a higher ORs of GH except in subjects with a high FPG level, who had the highest mean ACR level. Although our study was cross-sectional, we have demonstrated a potential role for hypertriglyceridemia in CKD.

At the single-nephron level, hyperfiltration is thought to represent an early link in the chain of events leading from intraglomerular hypertension to albuminuria and, subsequently, to a reduced GFR [6]. Experimental studies have demonstrated GFR measurement at the single-nephron level; however, this protocol is complicated and time-consuming, and is difficult to apply to a population study. Rather, population studies generally use measurements such as the renal clearance of iothalamate, Cr^51^-EDTA, or iohexol to evaluate the whole-kidney GFR, or calculate the eGFR using serum creatinine or cystitis C levels [1]. However, as nephron numbers vary by sex and decrease with age and progression of kidney disease [24], we defined GH as a value above the age- and sex-specific 95th percentile after excluding patients with an eGFR <60 ml/min/1.73 m^2^ or ACR ≥30 mg/g. However, some subjects with a normal whole-kidney filtration rate and no microalbuminuria might exhibit hyperfiltration at the single-nephron level. The loss of functional nephrons with progressive kidney might appear as normal GFR, while the remaining nephron might be in a state of hyperfiltration. In our study, although we expected a higher prevalence of GH with a systolic blood pressure ≥130 mmHg or FPG ≥140 mg/dL and serum triglyceride ≥500 mg/dL, we observed a relatively low prevalence of GH in this population, despite the highest ACR level. These results suggest that functional nephron loss can be explained by overlapping risk factors.

This study had several limitations. First, the design was cross-sectional, and therefore we can only prove correlation, but not causation. Therefore, longitudinal studies are needed to determine whether GH, when associated with the above-mentioned risk factors, is in fact a risk factor for renal injury in the general population. Second, GFR estimates at higher levels of estimated GFR are less accurate [25], which could be explained by larger effect of the inter-laboratory, biologic and measurement variability at higher GFR levels. Thus, there is the possibility to misclassify patients into the wrong category. In our study, there were some effort to minimize these variabilities although they were not perfect. First, creatinine levels were measured in a central laboratory with standardization of assays to minimize inter-laboratory variability. Second, to minimize the biologic variability, we used the CKD-EPI equation which is more accurate in persons with eGFR ≥60 mL/min/1.73 m2 compared with the MDRD Study equation. Third, to minimize the measurement variability, we measured the creatine level adjusting with the rate-blanked and compensated kinetic Jaffe method. Third, the methods used to measure eGFR may have been influenced by non-GFR factors, such as body composition. Although we adjusted our analysis for body weight, BMI, and waist circumference, we did not use a gold-standard method such as dual-energy X-ray absorptiometry, computed tomography, or magnetic resonance imaging. Forth, we defined GH by eGFR above the 95th percentile of age and sex matched healthy group. It was based on the age-related decline of GFR, but this definition also ignored that hyperfiltration also can happen in a single nephron, with globally decreased GFR and the possibility that elder people with GH by our definition could be healthy. Fifth, we adjusted antihypertension medication in multivariate analysis, but some kinds of antihypertension can influence on GH, independently blood pressure. Sixth, this study did not consider insulin resistance, despite a reported association of this factor with GH [26, 27], although this reported association is still controversial[28].

## Conclusion

Despite an increasing awareness of GH as a precursor to chronic kidney disease, previous studies have only included specific subpopulations. Therefore, we estimated the association of GH and other risk factors in an age- and sex-specific analysis of a dataset from a national representative population-based study (KNHANES). We found that in this sample, GH was associated with obesity, hyperglycemia, hypertension, hypertriglyceridemia, alcohol consumption and smoking all of which are known to associate with increased risk of CKD. We also found that GH associated independently with the ACR level. Paradoxically, however, subjects with diabetes and serum triglyceride level ≥500 mg/dL had a low prevalence of GH but the highest mean ACR level. Longitudinal studies are needed to further explore the risks of GH and microalbuminuria in a general population.

## Supporting information

S1 Table Demographic and clinical characteristics of healthy subjects. (DOCX)

S2 Table The estimated glomerular filtration rates (ml/min/1.73 m^2^) of healthy subjects stratified by age and sex. (DOCX)

S3 Table Multiple logistic regression analyses of odds ratios for hyperfiltration, stratified by fasting plasma glucose and serum triglyceride levels. (DOCX)
