# Supplementary material for: Association between metabolic parameters and glomerular hyperfiltration in a representative Korean population without chronic kidney disease
Source: PLoS One. 2018 Dec 6;13(12):e0207843. doi: 10.1371/journal.pone.0207843 (PMC6283579; doi:10.1371/journal.pone.0207843)
Supplement: S1 Table — (DOCX) [file pone.0207843.s001.docx]

**Table. Demographic and clinical characteristics of healthy subjects.**

| Phenotype | Value |
| --- | --- |
| N | 5907 |
| Age, years | 41.5±14.1 |
| Male gender, % | 42.1 |
| Weight, kg | 59.5±10.3 |
| Height, m | 162.7±8.5 |
| BMI, kg/m2 | 22.4±3.0 |
| SBP, mm Hg | 105±8 |
| DBP, mm Hg | 69±6 |
| HbA1c, % | 5.47±0.32 |
| Fasting plasma glucose, mg/dL | 88.6±5.9 |
| Total cholesterol, mg/dL | 184±33 |
| Triglyceride, mg/dL | 101±71 |
| HDL-cholesterol, mg/dL | 56±13 |
| Serum creatinine, mg/dL | 0.78±0.15 |
| eGFR by CKD-EPI equation, ml/min/1.73 m^2^ | 102±15 |
| Urine creatinine, g/dL | 1.66±0.89 |
| Urine albumin, mg/dL | 0.096±0.290 |
| ACR, mg/g | 0.561±1.440 |
| Energy intake, kcal | 2013±903 |
| Protein intake, g | 73.2±44.3 |
| Fat intake, g | 46.8±35.5 |
| Carbohydrate intake, g | 312±135 |
| Sodium intake, mg | 4430±3185 |

BMI, body mass index; SBP, systolic blood pressure; DBP, diastolic blood pressure; HbA1c, glycated hemoglobin; HDL, high-density lipoprotein; eGFR, estimated glomerular filtration rate; ACR, albumin creation ratio
