# Supplementary material for: Association between metabolic parameters and glomerular hyperfiltration in a representative Korean population without chronic kidney disease
Source: PLoS One. 2018 Dec 6;13(12):e0207843. doi: 10.1371/journal.pone.0207843 (PMC6283579; doi:10.1371/journal.pone.0207843)
Supplement: S2 Table — (DOCX) [file pone.0207843.s002.docx]

**Table. The estimated glomerular filtration rates (ml/min/1.73 m^2^) of healthy subjects stratified by age and sex.**

| Sex | Age (years) | N | eGFR (ml/min/1.73 m2) | | |
| --- | --- | --- | --- | --- | --- |
|  |  |  | Mean | Std. Deviation | 95th percentile |
| Men | 18–30 | 479 | 108.9 | 12.3 | 127.0 |
|  | 31–40 | 492 | 100.7 | 10.8 | 116.7 |
|  | 41–50 | 344 | 93.7 | 11.8 | 109.8 |
|  | 51–60 | 233 | 90.0 | 10.0 | 104.0 |
|  | 61–70 | 156 | 84.0 | 9.3 | 96.4 |
|  | >70 | 117 | 79.2 | 9.0 | 91.8 |
|  | Total | 1816 | 97.3 | 14.4 | 121.7 |
| Women | 18–30 | 915 | 117.0 | 11.0 | 130.9 |
|  | 31–40 | 1260 | 108.1 | 11.1 | 121.8 |
|  | 41–50 | 917 | 99.8 | 10.4 | 112.7 |
|  | 51–60 | 635 | 93.2 | 9.7 | 105.6 |
|  | 61–70 | 254 | 86.5 | 9.6 | 99.2 |
|  | >70 | 110 | 82.5 | 8.8 | 92.7 |
|  | Total | 4091 | 103.8 | 14.3 | 126.0 |
