# Supplementary material for: Association between metabolic parameters and glomerular hyperfiltration in a representative Korean population without chronic kidney disease
Source: PLoS One. 2018 Dec 6;13(12):e0207843. doi: 10.1371/journal.pone.0207843 (PMC6283579; doi:10.1371/journal.pone.0207843)
Supplement: S3 Table — (DOCX) [file pone.0207843.s003.docx]

Table. Multiple logistic regression analyses of odds ratios for hyperfiltration, stratified by fasting plasma glucose and serum triglyceride levels.

| Fasting plasma glucose (mg/dL) | Exp(B) | 95% CI | p-value |
| --- | --- | --- | --- |
| <100 | Ref. |  |  |
| ≥120 | 1.297 | 0.919–1.831 | 0.139 |
| ≥126 | 1.474 | 1.006–2.162 | 0.047 |
| ≥130 | 1.628 | 1.092–2.427 | 0.017 |
| ≥140 | 1.980 | 1.304–3.006 | 0.001 |
| ≥150 | 1.820 | 1.124–2.948 | 0.015 |
| ≥160 | 1.880 | 1.117–3.163 | 0.017 |
| ≥170 | 2.148 | 1.221–3.780 | 0.008 |
| ≥180 | 1.898 | 0.994–3.624 | 0.052 |
| ≥190 | 2.136 | 1.088–4.193 | 0.027 |
| ≥200 | 1.968 | 1.009–3.837 | 0.047 |
| Triglyceride (mg/dL) | Exp(B) | 95% CI |  |
| Normal (<150) | Ref. |  |  |
| Borderline (150–199) | 0.866 | 0.645–1.162 | 0.428 |
| High (200–499) | 0.963 | 0.731–1.267 | 0.928 |
| Very high (≥500) | 2.983 | 1.741–5.110 | <0.001 |
| ≥350 | 1.242 | 0.773–1.994 | 0.370 |
| ≥400 | 1.690 | 1.006–2.837 | 0.047 |
| ≥450 | 2.082 | 1.207–3.592 | 0.008 |
| ≥500 | 2.407 | 1.350–4.295 | 0.003 |
| ≥550 | 2.939 | 1.622–5.324 | <0.001 |
| ≥600 | 3.486 | 1.848–6.576 | <0.001 |
| ≥700 | 6.441 | 3.293–12.602 | <0.001 |

CI, confidence interval

* The analyses were adjusted for age, sex, body weight, body mass index, waist circumference, current smoking, alcohol consumption systolic blood pressure, fasting plasma glucose, serum triglyceride, energy intake and antihypertensive and lipid lowering medication.
